# Critical care at the end of life: a population-level cohort study of cost and outcomes

**DOI:** 10.1186/s13054-017-1711-4

**Published:** 2017-05-31

**Authors:** Dipayan Chaudhuri, Peter Tanuseputro, Brent Herritt, Gianni D’Egidio, Mathieu Chalifoux, Kwadwo Kyeremanteng

**Affiliations:** 1 University of Ottawa, Ottawa, ON Canada; 2 Bruyère Research Institute, Ottawa, ON Canada; 3 Ottawa Hospital Research Institute, Ottawa, ON Canada; 4 The Ottawa Hospital General Campus, 501 Smyth Road, Ottawa, ON K1H 8L2 Canada

**Keywords:** Palliative care, Terminal care, Costs and cost analysis

## Abstract

**Background:**

Despite the high cost associated with ICU use at the end of life, very little is known at a population level about the characteristics of users and their end of life experience. In this study, our goal was to characterize decedents who received intensive care near the end of life and examine their overall health care use prior to death.

**Methods:**

This was a retrospective cohort study that examined all deaths in a 3-year period from April 2010 to March 2013 in Ontario, Canada. Using population-based health administrative databases, we examined healthcare use and cost in the last year of life.

**Results:**

There were 264,754 individuals included in the study, of whom 18% used the ICU in the last 90 days of life; 34.5% of these ICU users were older than 80 years of age and 53.0% had more than five chronic conditions. The average cost of stay for these decedents was CA$15,511 to CA$25,526 greater than for those who were not admitted to the ICU. These individuals also died more frequently in hospital (88.7% vs 36.2%), and spent more time in acute-care settings (18.7 days vs. 10.5 days).

**Conclusions:**

We showed at a population level that a significant proportion of those with ICU use close to death are older, multi-morbid individuals who incur significantly greater costs and die largely in hospital, with higher rates of readmission, longer lengths of stay and higher rates of aggressive care.

**Electronic supplementary material:**

The online version of this article (doi:10.1186/s13054-017-1711-4) contains supplementary material, which is available to authorized users.

## Background

In Ontario, Canada, 98.9% of individuals access health care at least once in their last year of life costing the healthcare system, on average, CA$4.7 billion annually, or approximately 10% of the annual healthcare budget [1, 2]. Hospitalizations alone account for approximately 43% of those costs, with 20% of those costs as a result of at least one ICU visit. Current guidelines from the Society of Critical Care Medicine state that “in general, ICUs should be reserved for those patients with reversible medical conditions who have reasonable prospect of substantial recovery.” [3]. A prospective cross-sectional study conducted in a Canadian teaching hospital in 2014 suggested that in 37% of patients admitted to the ICU, at least one member of the care team believed that a patient was being given excessive or inappropriate care in the ICU [4]. Many studies suggest that advance care planning and palliative care interventions rather than ICU admissions in older, critically ill or dying patients reduce healthcare costs and provide patients with a better quality of life in the time they have left [3, 5–7]. Despite this evidence, in both the USA and Canada, ICU use has been increasing over time [1, 5].

In this study, we describe the use of ICU towards the end of life in Ontario, a province with over 13 million residents and near-universal health care [8]. Specifically, our goal was first to characterize decedents who received intensive care in the last few months, weeks and days of life. We then examined the costs associated with caring for those with ICU stays near the end of life, across a comprehensive set of healthcare sectors, including inpatient, outpatient and long-term care. It is expected - although never previously shown - that the cost associated with acute care stays among ICU users will dominate overall cost incurred across all sectors. Finally, we examined the dying experience of those with an ICU stay, including their location of death, rates of hospitalization and readmission, and rates of aggressive treatment. We compared this to the population that did not stay in the ICU.

While a few studies have examined ICU use at the end of life [9–11], this study, examined the ICU population and healthcare cost in greater detail - across a broad range of health sectors, and at a population level. Furthermore, it characterizes the typical ICU decedent in a large heterogeneous population and illustrates what their dying experience might be like, thus, allowing for possible areas of focus where palliative care and end-of-life planning can play a larger role in improving patients’ end-of-life outcomes.

## Methods

We conducted a retrospective cohort study examining healthcare use and cost incurred by decedents in their last 90 days of life. We captured all deaths in a 3-year period, from 1 April 2010 to 31 March 2013 (fiscal year (FY) 2010/11 to 2012/13) in Ontario, Canada. Using encrypted health card numbers as unique identifiers, records of healthcare use and costs were linked across various administrative databases. This study has been approved by the research ethics board at the Institute for Clinical Evaluative Sciences, at Sunnybrook Health Sciences Centre and at Ottawa Hospital Research Institute. No written consent was obtained; all data were encrypted using health card numbers as unique identifiers. Thus, all records used were de-identified and anonymized.

## Data sources and definitions

Deaths were identified using the Ontario Registered Persons Database (RPDB). The databases used to identify healthcare use are outlined in Additional file 1. We captured all decedents with a death date in the RPDB in our time period. We described the age and sex distribution of these individuals, the details being captured in the RPDB one year prior to death. The decedents’ socioeconomic status was measured using their neighborhood income one year prior to death. Following well-established methods, both neighborhood income and rurality were captured by linking to Statistics Canada census data using postal codes [12]. Decedents were further subdivided into quartiles using Aggregated Diagnosis Groups (ADG) and Adjusted Clinical Groups (ACG) scores. These algorithms were developed to predict the healthcare resource utilization that a patient may require as a result of their comorbidities, and have been shown to be strong predictors of one-year mortality in general ambulatory populations [13].

Intensive care use during a hospital admission in the last 3, 14, and 90 days of life were captured in the Canadian Institute for Health Information (CIHI) Discharge Abstract Database. We looked at the variables SCU-SCU6, which indicates codes when the patient is admitted into the ICU. Any of the non 9X codes occurring in the last 3, 14, and 90 days of life indicated that hospitalizations were ICU-related. We included stays in all types of ICU, including medical, surgical, and trauma ICUs. During these time periods, decedents were categorized as having no admission into hospital, having at least one admission but without an ICU stay, or having at least one admission with an ICU stay.

## Statistical analysis

All records of healthcare use were retrieved that had been paid for by the provincial Ministry of Health and Long Term Care (MOHLTC) in the last year of life. The cost associated with each record was estimated using costing methods developed for health administrative data described elsewhere [14]. Briefly, we have taken a payer (MOHLTC) costing perspective, using person-level healthcare expenditure that accounts for data for health care utilization and cost information per use. Cost information for sectors (e.g., hospitals, complex continuing care, rehabilitation) that have global budgets (e.g., by institution or by health region) are determined using a top-down approach through case-mix methodology. Sectors that have fee payments associated with each use (e.g., drug cost or cost paid out to the physician) have costs estimated directly. All costs were expressed in 2013 Canadian dollars; we inflated past costs using the healthcare-specific yearly Consumer Price Index reported by Statistics Canada. The health sector cost for the population was the sum of all costs among decedents captured within each respective sector. We also examined total cost within each sector by month prior to death.

All statistical tests were two-tailed and *p* = 0.05 was used to determine statistical significance. We used SAS 9.3 (SAS Institute Inc., Cary, NC, USA) for all analyses. We categorized our population by background demographic information such as age, sex, neighborhood income, rurality, primary core model, chronic conditions, and ADG quartile, and compared these across ICU users, non-ICU users, and all comers. Neighborhood income and rurality were derived from well-established methods from each decedent’s postal code of residence one year prior to death. Chronic conditions were derived from previously defined cohorts at the Institute for Clinical Evaluative Sciences, often based on previous records of healthcare use (e.g., physician claims, hospital visits, and medications), and at times validated. We then analyzed breakdowns of where costs occurred across different healthcare sectors, including acute (inpatient and emergency department), chronic (long-term care, complex, continuing care, home care, and rehabilitation) and outpatient care (clinics, physician billings, laboratory costs, drugs/devices, and non-physician OHIP billings) and compared these costs against ADG quartiles.

## Patient outcomes

We examined certain key health outcomes, such as location of death, length of stay in various places of care, readmission rates, cardiopulmonary resuscitation (CPR) rates and feeding tube insertion rates and examined how they differed amongst ICU users and non-ICU users. Location of death was determined by observing records of admissions in acute care, complex continuing care, long-term care, and rehabilitation facilities. All other deaths were classified as deaths in the community, and were broken down into those that occurred with or without home care support. The numbers of days spent in each of these settings were also observed in the last 90 days of life.

## Results

### General demographics

Overall, 264,754 individuals were included in the study, of whom 47,763 individuals (18%) were admitted to the ICU at least once in the last 90 days of life. The highest absolute numbers of ICU users were universally within the 80–89 years age range (Table 1). A larger percentage of ICU users lived in poorer neighborhoods and were rostered to a family physician. Individuals who stayed in the ICU at the end of life also had a greater disease burden, with a larger proportion in the third and fourth ADG quartiles when compared to the non-ICU-stay group (Table 1). Lastly, a larger proportion of individuals who stayed in the ICU had congestive heart failure (CHF), peripheral vascular disease (PVD), acute myocardial infarction, diabetes mellitus, asthma, stroke, or renal disease. Conversely, among the no-ICU population significantly larger proportions of individuals had dementia or cancer (Table 1).

**Table 1:** Characteristics and demographics of decedents in the last 90 days of life

	Number of decedents with ICU use in last 90 days of life (*n*, (%)) (N = 47,763)	Number of decedents with no ICU in last 90 days of life (*n*, (%)) (N = 216,991)	Total number of decedents (*n*, (%)) (N = 264,754)
Age, years			
0–19	1242 (2.6)	2841 (1.3)	4083 (1.5)
20–-39	1180 (2.5)	3798 (1.8)	4978 (1.9)
40–49	2054 (4.3)	6066 (2.8)	8120 (3.1)
50–59	5132 (10.7)	15,397 (7.1)	20,529 (7.8)
60-69	8852 (18.5)	26,594 (12.3)	35,446 (13.4)
70–79	12,836 (26.9)	42,626 (19.6)	55,462 (21.0)
80–89	13,625 (28.5)	75,332 (34.7)	88,957 (33.6)
90+	2842 (6.0)	44,337 (20.4)	47,179 (17.8)
Sex			
Female	20,919 (43.8)	11,4715 (52.9)	135,634 (51.2)
Male	26,844 (56.2)	102,276 (47.1)	129,120 (48.8)
Income quintile			
Lowest	11,212 (23.5)	48,853 (22.5)	60,065 (22.7)
Low	10,123 (21.2)	44,721 (20.6)	54,844 (20.7)
Middle	8964 (18.8)	41,507 (19.1)	50,471 (19.1)
High	8687 (18.2)	40,525 (18.7)	49,212 (18.6)
Highest	7902 (16.5)	38,544 (17.8)	46,446 (17.5)
Missing	875 (1.8)	2841 (1.3)	3716 (1.4)
Chronic conditions			
Osteoarthiritis	23,278 (48.7)	106,178 (48.9)	12,9456 (48.9)
Arthiritis - other	2178 (4.6)	8243 (3.8)	10,421 (3.9)
Cancer	18,045 (37.8)	96,854 (44.6)	114,899 (43.4)
Arrythmia	13,728 (28.7)	47,338 (21.8)	61,066 (23.1)
Dementia	5790 (12.1)	69,566 (32.1)	75,356 (28.5)
Depression	9426 (19.7)	44,674 (20.6)	54,100 (20.4)
Osteoporosis	3258 (6.8)	18,448 (8.5)	21,706 (8.2)
Renal	16,520 (34.6)	46,413 (21.4)	62,933 (23.8)
Stroke	8349 (17.5)	32,992 (15.2)	41,341 (15.6)
PVD	18,579 (38.9)	67,080 (30.9)	85,659 (32.4)
Asthma	8895 (18.6)	32,340 (14.9)	41,235 (15.6)
CHF	20,856 (43.7)	68,005 (31.3)	88,861 (33.6)
COPD	14,711 (30.8)	51,372 (23.7)	66,083 (25.0)
Hypertension	36,370 (76.2)	159,720 (73.6)	196,090 (74.1)
Diabetes mellitus	20,113 (42.1)	73,022 (33.7)	93,135 (35.2)
AMI	5035 (10.5)	7765 (3.6)	12,800 (4.8)
ADG quartiles			
1st quartile	5069 (10.6)	61,633 (28.4)	66,702 (25.2)
2nd quartile	13,096 (27.4)	53,853 (24.8)	66,949 (25.3)
3rd quartile	14,314 (30.0)	52,896 (24.4)	67,210 (25.4)
4th quartile	15,284 (32.0)	48,609 (22.4)	63,893 (24.1)

*PVD* peripheral vascular disease, *CHF* congestive heart failure, *COPD* chronic obstructive pulmonary disease, *ADG* Aggregated Diagnosis Groups, *AMI *acute myocardial infarction

**Table 2:** Mean cost in last 90 days of life across health care sectors (2013 Canadian dollars)

	Decedents with ICU stay (CAD $) (*N* = 47 763)				Decedents without ICU stay (CAD $) (*N* = 216 991)				All decedents (CAD $) (*N* = 264 754)			
ADG RANK	Q1	Q2	Q3	Q4	Q1	Q2	Q3	Q4	Q1	Q2	Q3	Q4
Continuing Care Sectors												
Long-term Care	248.70	353.29	539.78	866.63	3123.18	2615.20	2591.00	2841.76	2904.73	2172.74	2154.14	2369.28
Complex Continuing Care	348.79	521.96	827.13	1246.48	854.85	1632.18	2108.96	2934.99	816.39	1415.01	1835.97	2531.08
Home Care	619.95	854.89	1035.64	1179.18	1002.95	1804.33	1953.47	1936.17	973.84	1618.61	1758.00	1755.09
Rehabilitation	199.12	474.00	604.16	846.28	90.75	281.94	395.07	611.45	98.98	319.51	439.60	667.62
Acute Care Sectors												
Inpatient	18793.73	27287.33	35475.67	43227.11	3282.07	9420.51	13390.41	17700.34	4460.87	12915.46	18094.00	23806.66
Emergency Department	924.27	1046.35	1111.73	1163.73	317.33	638.54	779.46	873.87	363.45	718.31	850.23	943.21
Outpatient Care Sectors												
Outpatient clinics	392.43	800.53	1361.90	1311.71	463.46	1020.97	1100.33	987.50	458.06	977.85	1156.03	1065.05
Physician Billings	3988.53	5040.29	6086.83	6879.41	996.51	2004.52	2458.43	2797.10	1223.89	2598.35	3231.19	3773.64
Non-physician Billings (OHIP)	17.52	22.07	29.61	42.02	94.66	88.91	93.69	104.83	88.80	75.84	80.04	89.81
Laboratory (OHIP)	32.11	49.58	56.68	63.35	40.15	57.52	65.97	77.46	39.54	55.97	63.99	74.09
Drugs/Devices	402.64	572.71	745.36	788.34	600.16	883.20	965.75	983.36	585.15	822.47	918.81	936.71
Total Costs	25967.80	37023.00	47874.49	57614.23	10866.04	20447.83	25902.55	31848.83	12013.70	23690.12	30582.01	38012.23

### Costs of ICU stay

**Fig. 1:**
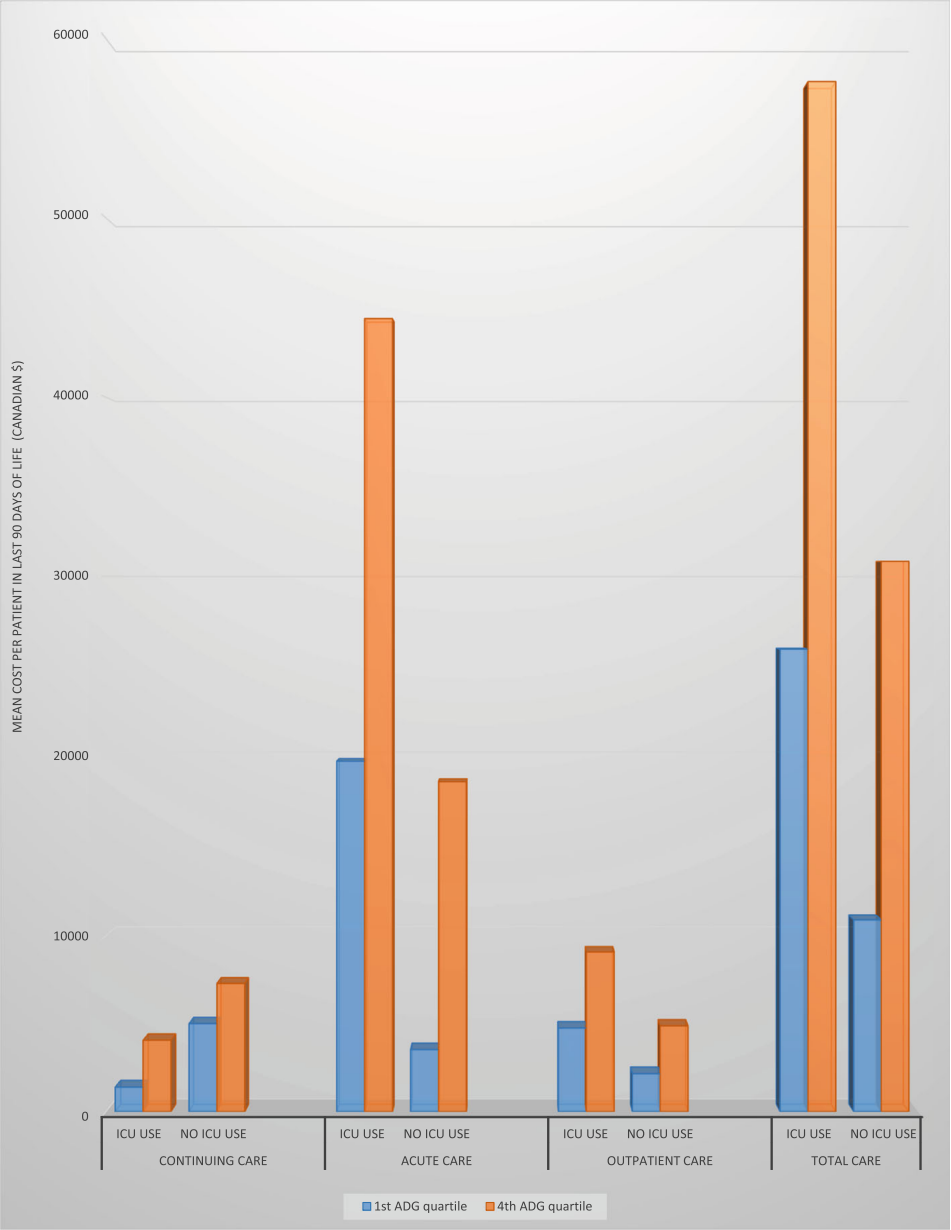
Healthcare costs in the last 90 days of life. Average healthcare cost per decedent in the last 90 days of life when distributed across healthcare sectors (continuing care, acute care and outpatient care), ICU use and burden of disease (represented by the first and fourth aggregated diagnosis group (*ADG*) quartiles). Patients in the first ADG quartile have the lowest burden of disease and those in the fourth ADG quartile have the highest burden of disease

Decedents who stayed in the ICU in the last 90 days of their life incurred much higher costs than those who did not (Fig. 1, Table 2). For example, in the first ADG quartile this amounted to an average cost difference of approximately CA$15,101 (139% increases) and in the fourth ADG quartile, the difference was CA$25,765 (81% increase). Most of this cost difference could be attributed to differences in inpatient costs - CA$15,511 (472% increase) in the first ADG quartile and CA$25,526 (144% increase) in the fourth ADG quartile (Table 2).

As the disease burden increased, the average cost across ADG quartiles in the ICU group increased by CA$31,646 (122%) and in the non-ICU group by CA$20,983 (193%). Most of this increase could be attributed to inpatient costs, with an average cost increase, across ADG quartiles, of CA$24,433 in the ICU group and CA$14,418 in the non-ICU group.

### Health outcomes

Individuals who were admitted to ICU in the last 90 days of life died in acute care by a far larger proportion than those who did not (88.7% vs. 36.2%) (Table 3, Table 4). Furthermore, they were readmitted to hospital more (40.22% vs. 16.89%) and 11.5% were readmitted to the ICU. They also spent more time in institutions (21.4 days vs. 14.8 days), specifically in acute care (18.7 days vs. 10.5 days). In addition, they also had higher rates of feeding tube insertions and CPR performed.

**Table 3:** Health outcomes of decedents in the last 90 days of life

	Number of decedents with ICU use in last 90 days of life (*n*, (%)) (N = 47,763)	Number of decedents with no ICU use in last 90 days of life (*n*, (%)) (N = 216,991)	Total number of decedents (*n*, (%)) (N = 264,754)
Location of death			
Acute care	42,381 (88.7)	78,602 (36.2)	120,983 (45.7)
Long term care	756 (1.6)	45,409 (20.9)	46,165 (17.4)
Complex continuing care	1746 (3.7)	18513 (8.5)	20,259 (7.7)
Home while receiving home care	1272 (2.7)	26,644 (12.3)	27,916 (10.5)
Rehabilitation	81 (0.2)	340 (0.2)	421 (0.2)
Other	1527 (3.2)	47,483 (21.9)	49,010 (18.5)
Readmission in last 90 days			
To hospital	19,211 (40.2)	36,649 (16.9)	55,860 (21.1)
To ICU	5491 (11.5)	N/A	5491 (2.1)
Feeding tube during hospitalization			
No	45,746 (95.8)	215,097 (99.1)	260,843 (98.5)
Yes	2017 (4.2)	1894 (0.9)	3911 (1.5)
Heart resuscitation during hospitalization			
No	42,265 (88.5)	214,667 (98.9)	256,932 (97.1)
Yes	5498 (11.5)	2324 (1.1)	7822 (3.0)

**Table 4:** Average length of stay in various places of care in the last 90 days of life

	Average length of stay amongst ICU users (days) (N = 47,763)	Average length of stay amongst non-ICU users (days) (N = 216,991)	Average length of stay amongst all decedents (days) (N = 264,754)
Places of care			
Acute care	18.7	10.5	12.0
Complex continuing care	1.3	3.3	2.9
Rehabilitation	0.6	0.3	0.4
Emergency department	0.8	0.7	0.7
*Total institution use*	21.4	14.8	16.0
Home care	7.7	11.4	10.8
*Total use*	29.1	26.3	26.8

## Discussion

Three important observations can be made as a result of this study. The first is that despite the general notion that a significant proportion of older, frail and multi-morbid individuals close to death do not do well in the ICU [15–18], we showed that at a population level, a significant proportion of those with ICU use when close to death are actually older and multi-morbid. Second, we showed the significant additional cost incurred by the healthcare system when decedents are admitted to an ICU, especially in relation to inpatient services. Last, we demonstrated that decedents with ICU use at the end of life die largely in hospital, with higher rates of readmission, longer lengths of stay, and higher rates of aggressive care including CPR and feeding tube insertion.

This is not the first study to examine use of ICU at the end of life. Angus et al. [9] conducted an analysis of ICU use in 1999 of all decedents in all non-federal hospitals in six states in the USA. They demonstrated similar rates of ICU use (22%) at the end of life, and when factoring in inflation they identified similar costs. Wunsch et al. [10] conducted a study examining differences in ICU use at the end of life between England and the USA. While the USA once again had similar ICU use to that identified in this study, the ICU use was significantly lower in England. Another study, performed in the Netherlands [11] compared terminally ill patients with and without cancer, showing that patients dying due to non-cancerous diseases were twice as likely to be admitted to the ICU. This study supports this body of evidence with more recent data and provides additional information on the differences in ICU use and cost, based on factors such as burden of disease. Furthermore, it provides much greater detail on the breakdown of costs at the end of life, and on the type of care provided at the end of life, to help identify areas where possible interventions could take place.

Many previous studies have shown that the elderly, with or without significant comorbidities often do worse in the ICU, both during their stay and after discharge [15–18]. This includes having increased mortality, increased morbidity, decreased quality of life, and decreased functional status [15, 16]. Our study also shows that a larger proportion of decedents who used the ICU had a greater disease burden when compared to decedents not using the ICU. While we cannot comment on the appropriateness of ICU admission in a descriptive retrospective study looking only at decedents, previous studies have shown that individuals with greater disease burden not only increase healthcare expenditures, but they also have higher mortality rates [13, 19–24], longer lengths of stay [23], higher readmission rates to the ICU in the same stay [22], and even perceive their quality of life to be worse after discharge from the ICU [21].

ICU care is also expensive, making up 0.5–1.0% of the Ontario gross domestic product (GDP) [25]. We clearly showed the significantly greater cost that ICU decedents incur at the end of life. Additionally, multiple studies show that most individuals do not wish for life-prolonging, intensive care towards the end of life [26–31]. While some elderly individuals can benefit from an ICU stay [18], this study suggests the need for careful thought and more research into appropriate ICU admissions at the end of life.

When it comes to end-of-life planning, most individuals prefer dying at home, with comfort care, rather than life-prolonging treatments [27–29]. Also, previous studies have shown that increased home care in the community leads to fewer hospitalizations and decreased costs in acute care [30]. Our study shows a significant discrepancy in location of death, length of hospital stay, place of stay, readmission rates, and rates of aggressive care amongst decedents who do and do not use the ICU. This may indicate potential missed opportunities for more dignified deaths through palliative care and early goals-of-life discussions.

There are a few limitations to this study [32]. First, since we only examined patients who died, we could not perform an unbiased analysis of whether patients are appropriately admitted to ICU at the end of life, as we did not have any information on the proportion of the population that survived. Thus, any conclusions made regarding the appropriateness of ICU admission based on a specific demographic or identifying characteristic can be biased either way, based on the outcomes of patients in that demographic who did not die. Second, in our broad population conclusions on the appropriateness of ICU admission and the quality of the end of life experience require additional details and may vary by subgroups within the population. For example, while the ICU group did have higher rates of death in an acute care setting and of aggressive care measures being performed, we cannot conclusively comment on whether this was appropriate without having additional information, such as the reason for admission, the underlying demographics, and the speed of disease progression in the patients who received these interventions. Nevertheless, given the large proportion of those admitted to ICU with advanced age and multiple comorbidities, there are potentially a significant number of inappropriate admissions and possible areas of intervention where healthcare costs could be reduced and the quality of the end of life experience may improve.

## Conclusions

In summary, our findings indicate that the majority of individuals admitted to ICU at the end of life are elderly and burdened by chronic illness. The existing medical literature indicates that this is the exact type of population that would be expected to do worse in the ICU [15, 20]. Moreover, end-of-life care costs are much more expensive when patients have a greater burden of disease and when they are admitted to the ICU. In addition, a large percentage of the population admitted to critical care at the end of life die in hospital with aggressive care measures taken. When put in the context of our rising healthcare costs [1] and a movement towards aging in place (in the community) [33, 34], this represents an area where more work is needed to ensure that only patients who can clearly benefit from critical care receive it at the end of life.

## Additional file

Additional file 1:Table S1. Characteristics and demographics of decedents in the last 90 days of life. **Table S2.** Databases used to record health care use and costs at the end of life. Description of all databases used to collect data for this study (DOCX 25 kb)
