# Supplementary material for: Critical care at the end of life: a population-level cohort study of cost and outcomes
Source: Crit Care. 2017 May 31;21:124. doi: 10.1186/s13054-017-1711-4 (PMC5450109; doi:10.1186/s13054-017-1711-4)
Supplement: Additional file 1:Table S1. — Characteristics and demographics of decedents in the last 90 days of life. Table S2. Databases used to record health care use and costs at the end of life. Description of all databases used to collect data for this study (DOCX 25 kb) [file 13054_2017_1711_MOESM1_ESM.docx]

Additional file 1

**Table S1:** Characteristics and Demographics of Decedents in the last 90 days of Life

|  | **ICU use in last 3 days of life** | **ICU use in last 14 days of life** | **ICU use in last 90 days of life** | **All Decedents with no ICU in last 90 days of life** | **All Decedents** |
| --- | --- | --- | --- | --- | --- |
| **Age** |  |  |  |  |  |
| 0-19 | 601 (4.38) | 902 (3.05) | 1242 (2.6) | 2841 (1.31) | 4083 (1.54) |
| 20-39 | 466 (3.4) | 829 (2.81) | 1180 (2.47) | 3798 (1.75) | 4978 (1.88) |
| 40-49 | 722 (5.26) | 1392 (4.71) | 2054 (4.3) | 6066 (2.8) | 8120 (3.07) |
| 50-59 | 1655 (12.07) | 3308 (11.2) | 5132 (10.74) | 15397 (7.1) | 20529 (7.75) |
| 60-69 | 2591 (18.89) | 5398 (18.28) | 8852 (18.53) | 26594 (12.26) | 35446 (13.39) |
| 70-79 | 3372 (24.59) | 7711 (26.11) | 12836 (26.87) | 42626 (19.64) | 55462 (20.95) |
| 80-89 | 3618 (26.38) | 8307 (28.13) | 13625 (28.53) | 75332 (34.72) | 88957 (33.6) |
| 90+ | 689 (5.02) | 1687 (5.71) | 2842 (5.95) | 44337 (20.43) | 47179 (17.82) |
|  |  |  |  |  |  |
| **Sex** |  |  |  |  |  |
| Female | 6088 (44.39) | 12962 (43.89) | 20919 (43.8) | 114715 (52.87) | 135634 (51.23) |
| Male | 7626 (55.61) | 16572 (56.11) | 26844 (56.2) | 102276 (47.13) | 129120 (48.77) |
|  |  |  |  |  |  |
| **Income quintile** |  |  |  |  |  |
| Lowest | 3282 (23.93) | 6995 (23.68) | 11212 (23.47) | 48853 (22.51) | 60065 (22.69) |
| Low | 2903 (21.17) | 6184 (20.94) | 10123 (21.19) | 44721 (20.61) | 54844 (20.72) |
| Middle | 2510 (18.3) | 5485 (18.57) | 8964 (18.77) | 41507 (19.13) | 50471 (19.06) |
| High | 2414 (17.6) | 5367 (18.17) | 8687 (18.19) | 40525 (18.68) | 49212 (18.59) |
| Highest | 2239 (16.33) | 4888 (16.55) | 7902 (16.54) | 38544 (17.76) | 46446 (17.54) |
| Missing | 366 (2.67) | 615 (2.08) | 875 (1.83) | 2841 (1.31) | 3716 (1.4) |
|  |  |  |  |  |  |
| **Rurality** |  |  |  |  |  |
| Missing | 290 (2.11) | 461 (1.56) | 638 (1.34) | 1873 (0.86) | 2511 (0.95) |
| Urban | 11439 (83.41) | 24950 (84.48) | 40578 (84.96) | 183401 (84.52) | 223979 (84.6) |
| Rural | 1985 (14.47) | 4123 (13.96) | 6547 (13.71) | 31717 (14.62) | 38264 (14.45) |
|  |  |  |  |  |  |
| **Primary Care Model** |  |  |  |  |  |
| Unrostered | 3394 (24.75) | 7061 (23.91) | 11126 (23.29) | 58626 (27.02) | 69752 (26.35) |
| Rostered | 10320 (75.25) | 22473 (76.09) | 36637 (76.71) | 158365 (72.98) | 195002 (73.65) |
|  |  |  |  |  |  |
| **Chronic Conditions** |  |  |  |  |  |
| Osteoarthiritis | 6236 (45.47) | 14073 (47.65) | 23278 (48.74) | 106178 (48.93) | 129456 (48.9) |
| Arthiritis - Other | 554 (4.04) | 1307 (4.43) | 2178 (4.56) | 8243 (3.8) | 10421 (3.94) |
| Cancer | 4170 (30.41) | 9850 (33.35) | 18045 (37.78) | 96854 (44.64) | 114899 (43.4) |
| Arrythmia | 3287 (23.97) | 7926 (26.84) | 13728 (28.74) | 47338 (21.82) | 61066 (23.07) |
| Dementia | 1381 (10.07) | 3363 (11.39) | 5790 (12.12) | 69566 (32.06) | 75356 (28.46) |
| Depression | 2510 (18.3) | 5596 (18.95) | 9426 (19.73) | 44674 (20.59) | 54100 (20.43) |
| Osteoporosis | 868 (6.33) | 1980 (6.7) | 3258 (6.82) | 18448 (8.5) | 21706 (8.2) |
| Renal | 4296 (31.33) | 9768 (33.07) | 16520 (34.59) | 46413 (21.39) | 62933 (23.77) |
| Stroke | 2574 (18.77) | 5384 (18.23) | 8349 (17.48) | 32992 (15.2) | 41341 (15.61) |
| PVD | 5059 (36.89) | 11341 (38.4) | 18579 (38.9) | 67080 (30.91) | 85659 (32.35) |
| Asthma | 2374 (17.31) | 5435 (18.4) | 8895 (18.62) | 32340 (14.9) | 41235 (15.57) |
| CHF | 4870 (35.51) | 11983 (40.57) | 20856 (43.67) | 68005 (31.34) | 88861 (33.56) |
| COPD | 3452 (25.17) | 8627 (29.21) | 14711 (30.8) | 51372 (23.67) | 66083 (24.96) |
| Hypertension | 9811 (71.54) | 22018 (74.55) | 36370 (76.15) | 159720 (73.61) | 196090 (74.06) |
| Diabetes | 5352 (39.03) | 11992 (40.6) | 20113 (42.11) | 73022 (33.65) | 93135 (35.18) |
| AMI | 1809 (13.19) | 3457 (11.71) | 5035 (10.54) | 7765 (3.58) | 12800 (4.83) |
|  |  |  |  |  |  |
| **Number of Chronic Conditions** |  |  |  |  |  |
| 0 | 832 (6.07) | 1292 (4.37) | 1658 (3.47) | 8024 (3.7) | 9682 (3.66) |
| 1 | 1023 (7.46) | 1857 (6.29) | 2717 (5.69) | 15850 (7.3) | 18567 (7.01) |
| 2 | 1563 (11.4) | 3058 (10.35) | 4604 (9.64) | 26096 (12.03) | 30700 (11.6) |
| 3 | 1967 (14.34) | 4046 (13.7) | 6266 (13.12) | 34493 (15.9) | 40759 (15.4) |
| 4 | 2043 (14.9) | 4468 (15.13) | 7192 (15.06) | 36321 (16.74) | 43513 (16.44) |
| 5 | 2029 (14.8) | 4459 (15.1) | 7396 (15.48) | 32706 (15.07) | 40102 (15.15) |
| 6 | 1720 (12.54) | 4039 (13.68) | 6758 (14.15) | 26166 (12.06) | 32924 (12.44) |
| 7 | 1209 (8.82) | 3026 (10.25) | 5208 (10.9) | 18214 (8.39) | 23422 (8.85) |
| 8 | 782 (5.7) | 1887 (6.39) | 3332 (6.98) | 10875 (5.01) | 14207 (5.37) |
| 9 | 348 (2.54) | 904 (3.06) | 1690 (3.54) | 5313 (2.45) | 7003 (2.65) |
| 10+ | 198 (1.44) | 498 (1.69) | 942 (1.97) | 2933 (1.35) | 3875 (1.46) |
|  |  |  |  |  |  |
| **ADG Quartiles** |  |  |  |  |  |
| 1st quartile | 2335 (17.03) | 3960 (13.41) | 5069 (10.61) | 61633 (28.4) | 66702 (25.19) |
| 2nd quartile | 4517 (32.94) | 9040 (30.61) | 13096 (27.42) | 53853 (24.82) | 66949 (25.29) |
| 3rd quartile | 3713 (27.07) | 8575 (29.03) | 14314 (29.97) | 52896 (24.38) | 67210 (25.39) |
| 4th quartile | 3149 (22.96) | 7959 (26.95) | 15284 (32) | 48609 (22.4) | 63893 (24.13) |

**Table S2**

Databases used to record health care use and cost at the end-of-life.

The Registered Persons Database (RPDB) is a population-based registry that is maintained by the Ministry of Health and Long-Term Care (MOHLTC) in Ontario, Canada. It contains records for all individuals registered to receive health services in Ontario. This database has demographic information including an individual’s date of birth, sex, address, date of death (where applicable), and captures changes in eligibility for health insurance coverage.

| **Health care Sector** | **Database** | **Description** |
| --- | --- | --- |
| **Continuing Care** |  |  |
| Long-term Care | Continuing Care Reporting System (CCRS) | Population-based resident information for over 600 publicly funded residential care homes with 24-hour nursing care |
| Complex Continuing Care | CCRS | Population-based information for all patients staying in a designated complex continuing care bed. These individuals are typically deemed to be in a non-acute  state, but still in need for treatment (e.g., rehabilitation) in an institution |
| Home Care | Home Care Database (HCD) Resident Assessment Instrument-Home Care (RAI-HC) | Data from the Ontario Association of Community Care Access Centers, responsible for providing all publicly funded home care |
| Rehabilitation | National Rehabilitation Reporting System (NRS) | Data from participating adult inpatient rehabilitation facilities and programs across Ontario |
| **Acute Care** |  |  |
| Inpatient without ICU | CIHI-DAD* | Administrative, clinical, and demographic data on all hospital discharges in Ontario |
| Inpatient with ICU | CIHI-DAD | Individuals with at least one Intensive Care Unit (ICU) visit in their last year of life |
| Emergency Department | National Ambulatory Care Reporting System (NACRS) | Captures all emergency department visits in Ontario |
| **Outpatient Care** |  |  |
| Outpatient clinics | NACRS | Select outpatient visits held in hospitals, including dialysis clinics and cancer care clinics |
| Physician Billings | Ontario Health Insurance Plan (OHIP) Claims Database | Claims data for physicians in Ontario—includes claims in both inpatient and outpatient settings. |
| Non-physician Billings | OHIP | Health professionals for provincially insured services, such as select midwives, oral surgeons, chiropractors, optometrists, and physiotherapists. Some care may occur for inpatients |
| Laboratory | OHIP | Outpatient laboratory services. Does not include laboratory services for inpatients |
| Drugs/Devices | Ontario Drug Benefit (ODB), Assistive Devices Program (ADP) | Drugs for those over 65 years, on social assistance, residents of LTC, home care recipients, Trillium drug program and special drugs program recipients for those qualifying for assistance. Select medically-necessary devices including home oxygen and respiratory devices. |

*****CIHI-DAD: Canadian Institute for Health Information-Discharge Abstract Database

Reproduced with permission from “the health care cost of dying: a population-based retrospective cohort study of the last year of life in Ontario, Canada”^1^
